# Multiple psychotherapeutic approaches and perspectives on eco-anxiety

**DOI:** 10.3389/fpsyg.2023.1162616

**Published:** 2023-04-18

**Authors:** Paolo Raile

**Affiliations:** Faculty Psychotherapy Science, Institute for Psychoanalytical-Ethnological Catastrophe Research, Sigmund-Freud Private University, Vienna, Austria

**Keywords:** eco-anxiety, psychotherapy science, approach-pluralism, treatment-expanding-psychotherapy-science, climate change

## Abstract

In highly diverse psychotherapy practices, psychotherapists with their individual schemas and personalities treat patients who are just as individual, each with his/her own partially dysfunctional schema, personality, worldview, and life situation. Intuition gained through experience is often applied, and a wide range of perspectives, techniques, and treatment options appropriate to the specific situation and psychotherapist-patient relationship are required for successful treatment of eco-anxiety manifestations. Several examples will be used to present the approaches of different psychotherapeutic approaches to eco-anxiety such as analytical psychology, logotherapy and existential analysis, psychodrama, and Morita-therapy. The treatment-possibilities-expanding psychotherapy science is presented, which helps psychotherapists to look beyond their original learned approach and learn about new perspectives and treatment methods in a methodologically sound way, which they already do intuitively.

## 1. Introduction

The climate crisis fundamentally threatens human existence and will be with us for most likely several centuries, even if the global community initiates immediate and comprehensive countermeasures. Since it does not currently appear that what needs to be done will be done, the crisis is likely to worsen, as demonstrated by an increase in extreme weather conditions and natural disasters, including crop failures. Increased worldwide morbidity and mortality resulting from heat waves, severe winters, floods, and droughts causing food and water shortages lead to social unrest and mass migration (IPCC, 2021, 2022). Personal experience, news, and social-media reports have aroused justified anxiety. Among experts, fears and other emotions related to the climate crisis have various names, such as eco-anxiety, eco-fear, climate anxiety, climate angst, environmental anxiety, and solastalgia (Coffey et al., 2021). These terms are used synonymously by some authors, while others differentiate them. Pihkala (2020a), for example, claims that climate anxiety is a special form of eco-anxiety that defines the field too narrowly and does not adequately describe the life situation of those affected.

Only the term eco-anxiety will be used herein for two reasons:

1. It is a common term used in scientific papers.

2. It describes the content more accurately than eco-fear, climate anxiety, or Glenn Albrecht’s (2005) neologism Solastalgia. The author defines eco-anxiety based on the prior work of several authors in this field, such as Clayton (2020), Clayton and Karazsia (2020), Hickman (2020), Hickman et al. (2021), Orange (2016), Pihkala (2020a,b), Raile and Rieken (2021), Raile (2023), and Taylor (2020) as the long-term fear of the uncertain consequences of the climate crisis and other relevant environmental impacts that have a clearly negative effect on the global and/or regional animate and inanimate ecological systems. It is closely related to eco-fear, the fear of concrete impacts of the climate crisis, as well as to eco-worry, which denotes a comparable, but weaker, feeling, whereas some researchers claim that worry is a different feeling which is connected to a third defense system next to fear and anxiety (Ojala et al., 2021). Eco-anxiety does not usually occur singularly, but often in combination with other eco-emotions, like sadness, despair, powerlessness, anger, guilt, shame, and grief (Cunsolo et al., 2020). It would be too short-sighted to consider eco-anxiety as the emotional state of a single individual. A societal perspective must be included since the triggers are usually not natural events alone. Reports in mass media and social networks about the catastrophic effects of the climate crisis and other anthropogenic environmental problems, as well as dissatisfaction with the inactivity of relevant decision makers and greater parts of the population, intensify public anxiety (Crandon et al., 2022). Eco-anxiety is usually an adequate and non-pathological reaction to a real threat. But it can, if it becomes too intense, significantly limit quality of life and everyday functionality. In such cases, psychotherapeutic treatment may be indicated. Eco-anxiety has only been discussed to a limited extent in the psychotherapeutic literature, although the number of scientific texts treating the phenomenon from different perspectives, such as psychology, sociology, and psychotherapy, has increased considerably in recent years (Pihkala, 2020a; Hickman et al., 2021; Thoma et al., 2021; Doherty et al., 2022).

Psychotherapy is not a homogeneous field of psychological interventions, but a combination of diverse approaches (Corsini, 1994), which is advantageous. There are numerous therapeutic approaches, theories, methods, and techniques for the countless different therapist-patient constellations. The author takes a constructivist point of view and claims that every therapeutic relationship is a unique combination of one or more therapists with their own schemes and personalities and one or more patients with their own sets of schemes, which may be dysfunctional. The constructivist viewpoint, as well as examples of different psychotherapeutic approaches and how they deal with aspects of eco-anxiety that occur in practice, is presented in the following two chapters. The conclusion is a scientifically based plea for a methodological, pluralistic approach to eco-anxiety in the field of psychotherapy. First, the chosen method, TEP based on radical constructivism, is introduced. Next, several chosen case examples are discussed in relation to eco-anxiety: Verena Kast’s analytic psychology, Elisabeth Lukas’ logotherapy, psychodrama according to Stadler and Kern, and Morita Therapy. These case examples consist of two reality-based parts, but the combination is fictious. The patients and their data are real but pseudonymized (with the knowledge and consent of the patients), and the treatments and the patients’ responses to those treatments are based on the writings of the presented authors.

## 2. Treatment-possibilities-expanding psychotherapy science

Psychotherapeutic science focuses on psychotherapy in all its aspects, including different treatment approaches, theories, methods, techniques, and interventions, as well as all their applications in areas that do not serve the curative treatment of people with mental issues, e.g., the interpretation of cultural phenomenons. Several attempts to establish a (general) science of psychotherapy have been undertaken, such as Grawe et al. (1994), Grawe (2004), Petzold (1993), and Fischer (2008, 2011). These are mainly based on a specific psychotherapeutic or an integrative approach. Diametrically contrasted is Greiner’s science of experimental psychotherapy (Greiner, 2012, 2020), which is based on Wallner’s theoretical constructive realism (Wallner, 1992). It is the only psychotherapeutic approach that enters the field from without *via* the philosophy of science. The author also follows a comparable path, which is not constructive realism, but an enhanced radical constructivism according to Glasersfeld (1987, 1996) as described in Raile (2022, 2023). The core assumption is that human reality is a construct with which one can act purposefully or viably in the world, i.e., by achieving the goals of thoughts and actions. Knowledge is a collection of perceptual patterns and actions, so-called schemas, which are formed according to the criterion of viability and Piaget’s mechanisms of accommodation and assimilation. Scientific knowledge can only be distinguished gradually from everyday knowledge. It is regarded as more reliable than our everyday knowledge, not because it is constructed in any special way, but because it is explicit and repeatable. The value of scientific knowledge does not depend on its truth in the philosophical sense, but on its viability (Glasersfeld, 1996).

Power structures and intersubjective comprehensibility are also relevant in the field of science. If scientific knowledge is communicated by statements in a book or by teaching and is regarded as viable, we speak of second-order viability or intersubjective viability. Whether such knowledge/statements are written and taught also depends on the power structures in the respective discourse (Foucault, 1982). There are many examples of the effects of such power structures, especially in the field of psychotherapy. The numerous resignations, expulsions, and newly founded schools exemplify the controversial and occasionally productive disputes within that scientific community. The reasons are often the adaption of a scientific construct from one person, for example Freud’s psychoanalysis, which proved to be viable for him, but not for another person, like his early follower, Alfred Adler. The latter modified the concept in a way that led to greater viability in his own world-construct. Freud did not consider Adler’s adaption as viable. Adler’s position was ostracized within the psychoanalytic discourse due to the scientific community’s power structures. As a result, Adler left the Viennese Psychoanalytic Association, founded his own school, and established new knowledge and new power structures in which his concept was considered viable (Handlbauer, 1998). The future psychoanalysts and psychologists matured and perpetuated such structures, which led to many new psychotherapeutic approaches. This explains today’s approach- and school-specific structures, including acceptance of and resistance to other approaches.

Whoever completes psychotherapeutic training anywhere today must choose a specific therapeutic approach, whereby only those may be chosen which are recognized by the respective national laws. S/he will only be qualified to work as a psychotherapist after learning the concept and the school-specific knowledge structures (Pritz, 2002). Psychotherapy as viewed from a constructivist perspective is practiced on a wide variety of patients, each with his/her own worldview, which is often dysfunctional in some areas. These patients are treated by no less diverse psychotherapists with their own worldviews. All people continue to develop and adapt their schemata. This not only refers to their personal schemata, which change with increasing life and psychotherapy experience, but also the professional-scientific ones of psychotherapists. The latter learn new techniques, improvise (as Perls, 1985, p. 170 stated, “…a good Gestalt psychotherapist no longer uses techniques, but is flexible and improvises.”), try out interventions, and develop new ideas and hypotheses, whereby their therapeutic methods change as they adapt scientific schemas. When they publish their viable schemes, others can be inspired to put them into practice themselves. But how can they know which methods are viable for which patients and when, if everything is in flux, and every person is different? Randomized controlled studies (RCTs) are not very helpful because they never reflect the individual patient’s worldview and his or her own schemas (Köhlke, 1992; Kriz, 2019). The answer is intuition, i.e., acting spontaneously and not consciously in a situation based on schemata acquired and adapted through experience. It is the basis of the choice of methods and techniques, but it also requires a certain flexibility, self-reflection, and a repertoire of alternative approaches, perspectives, and techniques (see Raile, 2023).

Raymond Corsini wrote his famous book “Current Psychotherapies” to show psychotherapists how other approaches work and to expand their knowledge and repertoire of techniques (Corsini, 1994). Treatment-expanding psychotherapeutic science (TEP) was created for the same reason, but represents a clear method based on an explicit theory (radical constructivism). It is an integrative method for practitioners. In TEP, a particular phenomenon, in this case eco-anxiety, is considered from the perspective of different psychotherapeutic approaches (Raile, 2023). For example, eco-anxiety is embedded in Verena Kast’s (1999, 2018) construct of analytical psychology or Lukas’ (2014) construct of logotherapy and existential analysis, and we look at how it is interpreted and treated there. The next chapter gives some short examples of such integrations.

## 3. Eco-anxiety from the perspective of different psychotherapeutic approaches

According to Verena Kast’s analytical psychology, emotionally stressful and traumatic experiences in a relationship, i.e., splintered and repressed, generalized and condensed relationships, can lead to complexes. These can be activated by present situations similar to these traumas. When a complex is activated, the person reacts with a stereotypic overreaction reflecting his/her life history. Severe anxiety can indicate such an activated complex if it occurs in certain situations (Kast, 1999, 2018). For example, a female patient experiences severe anxiety and reacts with uncontrolled outbursts of anger or crying when she reads in the media about disasters that claimed human and animal lives, such as during the Australian bushfires in 2020. She pities the baby koalas growing up without parents. In therapy, childhood memories are addressed, revealing an abandonment complex. When she was a toddler, her mother often left without a word despite the child’s crying and screaming and returned only after a long absence without explaining where she had been or what she had been doing. These relational, generalized and condensed experiences form the basis of the complex that is reactivated by thoughts and reports of abandoned babies or by dreams and fantasies of being the last survivor of a global climate catastrophe and thus abandoned by everyone. Through the technique *Active Imagination*, she can establish contact with the repressed part of her psyche, perceive the feelings she felt as a child, and put herself in her mother’s place. Over time, she becomes more and more successful in recognizing such situations and actively telling herself that she will not be abandoned, which does not make the anxieties go away, but at least reduces them to the point that they no longer overwhelm her. As shown here, these cases can include both real eco-anxiety and dynamics from personal history that activate and/or reinforce eco-anxiety.

An alternative approach taken by the Jungian psychotherapist Kast is called archetypes, which can be applied to eco-anxiety. These are structures in the collective unconscious which contain certain symbolisms that transcend cultures and eras, such as the circle, heroines, the divine child, ancient sages, the mysterious stranger, and so on (Kast, 1999, 2018). In the context of eco-anxiety, the archetypes of the nurturing mother nature and the world fire (apocalypse), the end of all existence, are especially relevant. The fact that mother nature, the principle of life, is seriously threatened by the climate crisis, and images of the end of the world are widespread, generates fear. Hope and confidence are conveyed by another archetype, namely that of the heroine, currently embodied by Greta Thunberg. Fears can be alleviated, e.g., by dreaming or fantasizing that one could save the world like superheroine Thunberg. This can also motivate people to become active for climate protection (Raile, 2023).

Another interesting approach which can be applied to eco-anxiety is logotherapy and existential analysis according to Elisabeth Lukas. Based on Frankl’s logotherapy, which states that the will to meaning and a meaning in life are of central importance, including the meaning of love, work, and suffering, Lukas states that so-called noogenic neurosis is rooted in suffering from a lack of meaning. An anxiety disorder, in contrast, is a psychogenic neurosis in Frankl’s terminology (Lukas, 2014). Lukas proposes the treatment of anxiety by means of self-distancing (Lukas, 2014), i.e., people with an anxiety problem very often think about their worries and problems and try to avoid certain situations. This is common, especially with social anxieties caused by, e.g., fear of criticism. Such anxiety can arise when one talks about one’s eco-anxiety and is often met with rejection and mockery instead of understanding (Hickman, 2020). One way out is paradoxical intention, in which individuals are guided to intentionally trigger the feared effect to escape from the fear spiral and experience that the consequences are not as bad as what was feared (Lukas and Wurzel, 2015).

In psychodrama according to Stadler and Kern, which is often applied in a group setting, protagonists are at the center of a group, set their stage, appoint auxiliary selves to support their role-play, and creatively work through a theme (Stadler and Kern, 2010). One treatment of eco-anxiety consists of re-enacting a scene in which the person at the kitchen table reads the news and is then insidiously attacked by climate anxiety, portrayed by a person from the group called an auxiliary ego. The news is also represented by a person who shouts out loud disaster messages. The auxiliary ego is introduced through role reversal, i.e., the protagonist takes on the role of the news or the fear and acts out that role. Afterwards, the roles are changed, and the auxiliary ego plays the patient’s role. During play, earlier memories of similar feelings can arise, which are also immediately replayed and lead to new insights. Psychodrama also strengthens resources like creativity, which positively influences problem-solving skills and has an additional effect that is particularly evident in the group (Stadler and Kern, 2010). After the end, the auxiliary egos report on their thoughts and feelings, e.g., the mounting fear meant that s/he was sad that the person who attacked him/her was paralyzed with fear instead of startled and activated. She only snuck up on them to keep them from running away. Otherwise, her action would have seemed like a “kick in the butt.” Such and other feedbacks open new perspectives on one’s experienced eco-anxiety. This is only one possible application of Psychodrama. Its wide variety of methods can be applied in numerous creative ways to eco-anxiety.

Morita Therapy, named after its founder Shoma Morita and represented by David Reynolds, focuses on mindfulness and acceptance of a person’s inevitable spontaneous thoughts and feelings while pointing out that these are uncontrollable and will subside over time, but actions are always controllable despite them. The immediate effect of therapy in an outpatient setting is acceptance of the feelings and thoughts as an unchangeable part of your nature, while still being able to act as necessary given the external circumstances even in the face of anxiety (Reynolds, 1994). The body and mind become accustomed to the climatic anxiety, and its intensity decreases.

## 4. Discussion

The case reports are partly reality based as explained in the last paragraph of the introduction and shows some possible treatment options of different approaches. They demonstrate in a practical way what is exemplified in some existing psychotherapeutic literature (Passmore and Howell, 2014; Pihkala, 2020a; Doherty et al., 2022; Raile, 2023). There are texts about existing approaches like CBT, psychoanalysis, or systemic therapy, and there are new approaches like ecotherapy which is related to analytical psychology according to Jung as well as to psychosynthesis according to Assagioli. It is important to point out that the practical demonstrations are only a few treatment options. There are many others like one part of ecotherapy, which claims that a strong connection with and being in nature helps coping with overwhelming eco-emotions (Raile, 2023).

These different therapeutic approaches illustrate very different ways of interpreting and treating eco-anxiety. The effects of the methods, techniques, and interventions can be summarized in seven groups that revolve around the following themes: revealing the anxiety-reinforcing factors hidden behind eco-anxiety, turning to something higher (meaning, spirituality, God, community, etc.), distancing oneself from feelings as anxiety, fear, grief, or anger, strengthening problem-solving resources, alleviating the feeling by counteracting the anxiety through humor, hope, or encouragement, changing the perspective, and confronting or exposing the anxiety (Raile, 2023; for emotion-focused methods, see Hamilton, 2022; Pihkala, 2022).

## 5. Conclusion

This article, based on radical constructivism, explains that psychotherapy practice is highly diverse. Psychotherapists with their individual schemas and personalities treat patients who are just as individual, each with his/her own partially dysfunctional schemas, personalities, and life-worlds. Successful treatment applies intuition gained through experience, and interventions or techniques appropriate to the specific situation and psychotherapist-patient relationship are chosen. Knowledge of and experience in a wide range of possible perspectives and (treatment) options is helpful when confronting the full spectrum of eco-anxiety manifestations. This is probably one of the reasons why psychotherapists look for new methods, approaches, and perspectives after completing their training in a particular approach (Crameri et al., 2018; Norcross and Alexander, 2019). This also presents an argument for maintaining the diversity of psychotherapeutic approaches because they significantly improve the quality of care, not only in the field of eco-anxiety. TEP can also help psychotherapists look beyond their originally learned approach and learn about new perspectives and treatments in a methodologically sound way, which they already do intuitively.

## Data availability statement

The original contributions presented in the study are included in the article/supplementary material, further inquiries can be directed to the corresponding author.

## Author contributions

The author confirms being the sole contributor of this work and has approved it for publication.

## Conflict of interest

The author declares that the research was conducted in the absence of any commercial or financial relationships that could be construed as a potential conflict of interest.

## Publisher’s note

All claims expressed in this article are solely those of the authors and do not necessarily represent those of their affiliated organizations, or those of the publisher, the editors and the reviewers. Any product that may be evaluated in this article, or claim that may be made by its manufacturer, is not guaranteed or endorsed by the publisher.
